# 4012 Positive Deviants for Medication Therapy Management: A Mixed-Methods Comparative Case Study of Community Pharmacy Practices

**DOI:** 10.1017/cts.2020.420

**Published:** 2020-07-29

**Authors:** Omolola A Adeoye-Olatunde, Leslie M. Lake, Karen S. Hudmon, Alan J. Zillich, Margie E. Snyder

**Affiliations:** 1 Indiana University School of Medicine; 2 Kroger Central Division; 3 Purdue University College of Pharmacy

## Abstract

OBJECTIVES/GOALS: To optimize medication use in older adults, Medication Therapy Management (MTM) was launched as part of Medicare Prescription Drug (Part D) policy. The objective of this study was to generate hypotheses for strategies that contribute to community pharmacies’ ability to achieve high performance on policy relevant MTM quality measures. METHODS/STUDY POPULATION: This mixed-methods comparative case study design incorporated two conceptual models; the Positive Deviance model and Chronic Care Model. The study population consisted of pharmacy staff employed by a Midwestern division of a national supermarket-community pharmacy chain. Data consisted of semi-structured interviews and demographics. Qualitative and quantitative data were analyzed abductively or using descriptive statistics, respectively. Case comparisons were synthesized using the Framework Method. MTM quality measures used to evaluate participant pharmacies’ MTM performance mirrored quality measures under Domain 4 (Drug Safety and Accuracy of Drug Pricing) of the 2017 Medicare Part D Plan’ Star Rating measures. RESULTS/ANTICIPATED RESULTS: Staff at 13 of the 18 selected pharmacies (72.2%) participated in interviews. Interviewees included 11 pharmacists, 11 technicians and three student interns. Strategies hypothesized as contributing to MTM performance included: 1. Strong pharmacist-provider relationships and trust, 2. Inability to meet patients’ cultural, linguistic, and socioeconomic needs (negatively contributing), 3. Technician involvement in MTM, 4. Providing comprehensive medication reviews in person vs. phone alone, 5. Placing high priority on MTM, 6. Using maximum number of clinical information systems (CISs) to identify eligible patients. 7. Technicians using CISs to collect information for pharmacists, 8. Faxing prescribers adherence medication therapy problems (MTPs) and calling on indication MTPs. DISCUSSION/SIGNIFICANCE OF IMPACT: Our study resulted in eight strategies hypothesized to contribute to community pharmacy performance on MTM quality measures. To inform MTM policy recommendations, future research should engage stakeholders to assist with prioritizing hypotheses to be tested in a larger representative sample of pharmacies. CONFLICT OF INTEREST DESCRIPTION: This research was supported, in part, with support from the Indiana Clinical and Translational Sciences Institute funded, in part by grant number TL1TR001107 from the National Institutes of Health, National Center for Advancing Translational Sciences, Clinical and Translational Sciences Award. Dr. Adeoye-Olatunde is a part-time employee and Dr. Lake is a full-time employee at the Midwestern division, national supermarket-community pharmacy chain, where study procedures were conducted. Dr. Snyder reports personal fees from Westat, Inc., outside the submitted work.

